# Altered Glucagon and GLP-1 Responses to Oral Glucose in Children and Adolescents With Obesity and Insulin Resistance

**DOI:** 10.1210/clinem/dgad728

**Published:** 2023-12-13

**Authors:** Sara Elizabeth Stinson, Ierai Fernández de Retana Alzola, Emilie Damgaard Brünner Hovendal, Morten Asp Vonsild Lund, Cilius Esmann Fonvig, Louise Aas Holm, Anna Elisabet Jonsson, Christine Frithioff-Bøjsøe, Michael Christiansen, Oluf Pedersen, Lars Ängquist, Thorkild I A Sørensen, Jens Juul Holst, Bolette Hartmann, Jens-Christian Holm, Torben Hansen

**Affiliations:** Novo Nordisk Foundation Center for Basic Metabolic Research, Faculty of Health and Medical Sciences, University of Copenhagen, 2200 Copenhagen, Denmark; Novo Nordisk Foundation Center for Basic Metabolic Research, Faculty of Health and Medical Sciences, University of Copenhagen, 2200 Copenhagen, Denmark; Novo Nordisk Foundation Center for Basic Metabolic Research, Faculty of Health and Medical Sciences, University of Copenhagen, 2200 Copenhagen, Denmark; The Children's Obesity Clinic, accredited European Centre for Obesity Management, Department of Pediatrics, Holbæk Hospital, 4300 Holbæk, Denmark; The Children's Obesity Clinic, accredited European Centre for Obesity Management, Department of Pediatrics, Holbæk Hospital, 4300 Holbæk, Denmark; Department of Biomedical Sciences, Faculty of Health and Medical Sciences, University of Copenhagen, 2200 Copenhagen, Denmark; Novo Nordisk Foundation Center for Basic Metabolic Research, Faculty of Health and Medical Sciences, University of Copenhagen, 2200 Copenhagen, Denmark; The Children's Obesity Clinic, accredited European Centre for Obesity Management, Department of Pediatrics, Holbæk Hospital, 4300 Holbæk, Denmark; Department of Clinical Medicine, Faculty of Health and Medical Sciences, University of Copenhagen, 2200 Copenhagen, Denmark; Novo Nordisk Foundation Center for Basic Metabolic Research, Faculty of Health and Medical Sciences, University of Copenhagen, 2200 Copenhagen, Denmark; The Children's Obesity Clinic, accredited European Centre for Obesity Management, Department of Pediatrics, Holbæk Hospital, 4300 Holbæk, Denmark; Novo Nordisk Foundation Center for Basic Metabolic Research, Faculty of Health and Medical Sciences, University of Copenhagen, 2200 Copenhagen, Denmark; Novo Nordisk Foundation Center for Basic Metabolic Research, Faculty of Health and Medical Sciences, University of Copenhagen, 2200 Copenhagen, Denmark; The Children's Obesity Clinic, accredited European Centre for Obesity Management, Department of Pediatrics, Holbæk Hospital, 4300 Holbæk, Denmark; Department of Biomedical Sciences, Faculty of Health and Medical Sciences, University of Copenhagen, 2200 Copenhagen, Denmark; Department for Congenital Disorders, Statens Serum Institute, 2300 Copenhagen, Denmark; Novo Nordisk Foundation Center for Basic Metabolic Research, Faculty of Health and Medical Sciences, University of Copenhagen, 2200 Copenhagen, Denmark; Center for Clinical Metabolic Research, Herlev-Gentofte University Hospital, 2900 Copenhagen, Denmark; Novo Nordisk Foundation Center for Basic Metabolic Research, Faculty of Health and Medical Sciences, University of Copenhagen, 2200 Copenhagen, Denmark; Novo Nordisk Foundation Center for Basic Metabolic Research, Faculty of Health and Medical Sciences, University of Copenhagen, 2200 Copenhagen, Denmark; Department of Public Health, Faculty of Health and Medical Sciences, University of Copenhagen, 1353 Copenhagen, Denmark; Novo Nordisk Foundation Center for Basic Metabolic Research, Faculty of Health and Medical Sciences, University of Copenhagen, 2200 Copenhagen, Denmark; Department of Biomedical Sciences, Faculty of Health and Medical Sciences, University of Copenhagen, 2200 Copenhagen, Denmark; Novo Nordisk Foundation Center for Basic Metabolic Research, Faculty of Health and Medical Sciences, University of Copenhagen, 2200 Copenhagen, Denmark; Department of Biomedical Sciences, Faculty of Health and Medical Sciences, University of Copenhagen, 2200 Copenhagen, Denmark; Novo Nordisk Foundation Center for Basic Metabolic Research, Faculty of Health and Medical Sciences, University of Copenhagen, 2200 Copenhagen, Denmark; The Children's Obesity Clinic, accredited European Centre for Obesity Management, Department of Pediatrics, Holbæk Hospital, 4300 Holbæk, Denmark; Novo Nordisk Foundation Center for Basic Metabolic Research, Faculty of Health and Medical Sciences, University of Copenhagen, 2200 Copenhagen, Denmark

**Keywords:** adolescent, child, glucagon, GLP-1, GIP, obesity

## Abstract

**Context:**

Pediatric obesity is characterized by insulin resistance, yet it remains unclear whether insulin resistance contributes to abnormalities in glucagon and incretin secretion.

**Objective:**

To examine whether fasting and stimulated glucagon, glucagon-like peptide-1 (GLP-1), and glucose-dependent insulinotropic polypeptide (GIP) concentrations differ between children and adolescents with obesity and insulin resistance (OIR), obesity and normal insulin sensitivity (OIS), and controls with normal weight (NW).

**Methods:**

80 (34 boys) children and adolescents, aged 7-17 years with OIR (n = 22), OIS (n = 22), and NW (n = 36) underwent an oral glucose tolerance test with measurements of serum insulin, plasma glucose, glucagon, total GLP-1, and total GIP. Homeostatic model assessment of insulin resistance (HOMA-IR), single point insulin sensitivity estimator (SPISE), Matsuda index, insulinogenic index (IGI), and oral disposition index (ODI) were calculated.

**Results:**

Fasting concentrations of glucagon and GLP-1 were higher in the OIR group, with no significant differences for GIP. The OIR group had higher glucagon total area under the curve (tAUC_0-120_) and lower GLP-1 incremental AUC (iAUC_0-120_), with no significant differences in GIP iAUC_0-120_. Higher fasting glucagon was associated with higher HOMA-IR, lower Matsuda index, lower SPISE, higher IGI, and higher plasma alanine transaminase, whereas higher fasting GLP-1 was associated with higher HOMA-IR, lower Matsuda index, and lower ODI. Higher glucagon tAUC_0-120_ was associated lower SPISE and lower Matsuda index, whereas lower GLP-1 iAUC_0-120_ was associated with a higher HOMA-IR, lower Matsuda index, and lower ODI.

**Conclusion:**

Children and adolescents with OIR have elevated fasting concentrations of glucagon and GLP-1, higher glucagon and lower GLP-1 responses during an OGTT compared to those with OIS and NW. In contrast, individuals with OIS have similar hormone responses to those with NW.

With the escalating prevalence of pediatric obesity worldwide, children and adolescents are faced with an increased risk of developing type 2 diabetes (T2D), hepatic steatosis, and cardiovascular complications (1). Yet not all children and adolescents with obesity develop cardiometabolic comorbidities. One explanation for this heterogeneity could be related to relative differences in fat distribution. When the subcutaneous adipose tissue capacity is exceeded, the lipids are directed towards the visceral adipose tissue and nonadipose tissues, leading to insulin resistance—a concept known as “the adipose expandability theory” (2). Insulin resistance of obesity is linked to the clustering of cardiometabolic risk factors, and may serve as a marker distinguishing differing levels of metabolic derangement (3).

Of particular interest to obesity are glucagon and the incretin hormones, glucose-dependent insulinotropic polypeptide (GIP) and glucagon-like peptide-1 (GLP-1). Glucagon is secreted from the pancreas (α-cells) during fasting (4). GIP is secreted mainly from the upper (K-cells) and GLP-1 from the lower (L-cells) intestinal tract upon nutrient intake (5). Abnormalities in the secretion of pancreatic and intestinal hormones are observed with obesity and may be related to the level of insulin resistance, raising questions about the role of these hormones in development of obesity during the period of childhood and adolescence.

Our group recently demonstrated that plasma concentrations of glucagon are elevated at fasting in children and adolescents with overweight and obesity, associating with worsened insulin resistance, higher liver fat content, dyslipidemia, and hypertension, but do not necessarily associate with hyperglycemia (6, 7). Moreover, children and adolescents with obesity display diminished glucagon suppression during an oral glucose tolerance test (OGTT) or an euglycemic–hyperinsulinemic clamp (EHC) (8, 9), dependent on the level of insulin resistance (8).

GLP-1 responses to oral glucose or fat are seemingly blunted in children and adolescents with obesity when compared with controls with normal weight (NW) (10-12). Earlier work from our group showed that at fasting, plasma concentrations of total GLP-1 were elevated in children and adolescents with overweight and obesity compared with population-based controls, associating with insulin resistance, hyperglycemia, higher concentrations of plasma alanine transaminase (ALT), dyslipidemia, and hypertension (13, 14).

No significant differences in GIP response during an OGTT or EHC have previously been described in children and adolescents with obesity (15-18).

It still remains unclear whether insulin resistance mediates the effects of obesity on impaired glucagon and gut hormone secretion, and whether this occurs early on in disease pathogenesis (19). The primary aim of this study was to evaluate whether plasma concentrations of glucagon, total GLP-1, and total GIP during an OGTT differ between children and adolescents with obesity and insulin resistance (OIR), obesity and normal insulin sensitivity (OIS), and controls with NW. Secondly, we aimed to examine the associations of these hormones with estimates of insulin sensitivity, pancreatic β-cell function, and fasting levels of liver enzymes. We hypothesize that children and adolescents with OIR will have elevated fasting concentrations of these hormones, and higher glucagon and lower GLP-1 and GIP responses during an OGTT, which will associate with worsened insulin sensitivity, β-cell function, and elevated liver enzymes.

## Materials and Methods

### Study Groups

This study includes 80 (34 boys) children and adolescents, aged 7-17 years, who participated in a standard OGTT. Participants were invited from the HOLBAEK study, which comprises an obesity group, the members of which underwent a multifaceted, holistic, childhood obesity management program at Holbæk Hospital (20) and a population-based group recruited from schools across Zealand, Denmark (21).

The HOLBAEK study, formerly known as the Danish Childhood Obesity Data and Biobank, is registered with ClinicalTrials.gov identifier number NCT00928473.

Exclusion criteria were monogenic forms of obesity (eg, pathogenic mutations in the Melanocortin 4 receptor [*MC4R*]), diagnosed type 1 diabetes or T2D, and screen-detected T2D (22) based on the blood samples taken during the OGTT (fasting glucose ≥7.0 mmol/L and/or 2-hour glucose ≥11.1 mmol/L; n = 1), and failure to complete the OGTT.

**Figure 1.**
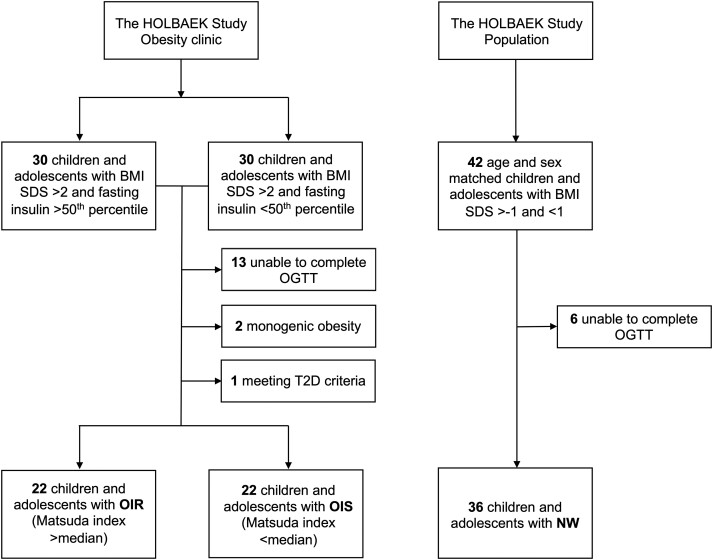
Study flow chart. BMI SDS, body mass index standard deviation score; NW, normal weight; OGTT, oral glucose tolerance test; OIR, obesity and insulin resistance; OIS, obesity and normal insulin sensitivity; T2D, type 2 diabetes.

Participants with a body mass index (BMI) standard deviation score (SDS) ≥ 2 were recruited from the HOLBAEK study obesity clinic cohort (23), and 30 + 30 were selected out of those with a historic fasting serum insulin concentration above or below the 50th age- and sex-specific percentiles, respectively (Fig. 1). Following exclusion of 16 participants, the remaining 44 participants were then regrouped based on the Matsuda index into 2 categories, OIR or OIS (Fig. 1). Forty-two age- and sex-matched participants with BMI SDS ≥ −1 and ≤1 were selected from the HOLBAEK study population cohort, following exclusion of 6 participants, leaving a total of 36 participants in the NW group (Fig. 1).

### Ethics

The study was conducted at the Children's Obesity Clinic, Department of Pediatrics, Copenhagen University Hospital at Holbæk, Denmark, from June 2013 to November 2013. The study was conducted according to the principles of the Declaration of Helsinki, and oral and written informed consent was obtained from all participants. Since the participants were younger than 18, consent was accepted if the parents also gave informed written consent. The study was approved by the Ethics Committee of Zealand Region (protocol no. SJ-104) and the Danish Data Protection Agency (REG-043-2013).

### Anthropometrics

Height was measured by stadiometer to the nearest 1 mm, and weight was measured to the nearest 0.1 kg on a Tanita medical scale (WB-110). Age- and sex-adjusted BMI SDS were calculated and evaluated according to a Danish reference (24).

### Oral Glucose Tolerance Test

Following a 10-hour overnight fast, participants underwent a 2-hour OGTT (1.75 g/kg, maximum 75 g). Blood samples were obtained at 0, 30, and 120 minutes for the measurement of plasma glucose, serum insulin, plasma glucagon, plasma total GLP-1, and plasma total GIP. Impaired fasting glucose (IFG) and/or impaired glucose tolerance (IGT) was defined based on fasting plasma glucose between 5.6 and 6.9 mmol/L and 2-hour plasma glucose between 7.8 and 11.0 mmol/L according to guidelines from the American Diabetes Association (22).

### Biochemical Analyses

Blood samples were immediately separated by centrifugation and stored at −80 °C until analysis. Concentrations of plasma glucose were determined by Siemens Dimension Vista and serum insulin by enzymatic calorimetric methods on a Cobas e 601, and plasma high-density lipoprotein cholesterol (HDL-C), plasma triglycerides (TG), plasma ALT, aspartate transaminase (AST), and plasma γ-glutamyl transferase (GGT) were measured on a Siemens Dimension Vista (25). Blood samples were collected in ice-cold EDTA vials for glucagon, GLP-1, and GIP measurements. Plasma concentrations of glucagon (Mercodia Cat# 10-1271-01, RRID:AB_2737304), total GLP-1 (Mercodia Cat# 10-1278-01, RRID:AB_2892202), and total GIP (Mercodia Cat# 10-1258-01, RRID:AB_2895085) were quantified by enzyme-linked immunosorbent assay (Mercodia, Uppsala, Sweden) in duplicate and run on a SpectraMax iD3 (San Jose, CA, USA).

### Calculations

Homeostatic model assessment of insulin resistance (HOMA-IR) was calculated as (fasting insulin mU/L × fasting glucose mmol/L)/22.5 as an estimate of hepatic insulin resistance (26). The single-point insulin sensitivity estimator (SPISE) was calculated as 600 × HDL-C^0.185^/(TG^0.2^ × BMI^1.338^) as a marker of whole-body insulin sensitivity (27). The Matsuda index was calculated as 10 000/√([fasting glucose × fasting insulin] × [mean glucose × mean insulin during OGTT]), representing a composite of both hepatic and peripheral tissue sensitivity to insulin (28). The insulinogenic index (IGI) was calculated as (insulin_30_ – insulin_0_)/(glucose_30_ – glucose_0_) (29), which has been shown to correlate with acute insulin response (30). The oral disposition index (ODI) was calculated as (Δinsulin_0 − 30_/Δglucose_0 − 30)_ × (1/fasting insulin) as an estimate of β-cell function (31).

Total area under the curve (tAUC_0-120_) and incremental AUC (iAUC_0-120_) were calculated for glucose, insulin, total GLP-1, and total GIP using the trapezoidal method. Relative AUC (rAUC_0-120_) was calculated as tAUC_0-120_/(fasting concentration × 120 minutes).

### Statistical Analyses

Data were tested for normal distribution by the Shapiro–Wilk test and for equal variance by Levene's test. Data are presented as mean (SD) or median (interquartile range) for continuous, normally, and non-normally distributed variables, respectively, and frequencies and percentages for categorical variables. Differences between the groups were evaluated using Kruskal–Wallis tests for continuous variables (post hoc pair-wise comparisons using Wilcoxon rank sum tests with Bonferroni correction to adjust for multiple testing) and χ^2^ test for categorical variables.

Linear regression was applied to test the associations between fasting hormones and hormone response during the OGTT (tAUC, iAUC, rAUC) on HOMA-IR, SPISE, Matsuda index, IGI, ODI, and fasting levels of liver enzymes (ALT, AST, GGT) in a pooled model with all individuals, adjusted for age, sex, BMI SDS. A further subanalysis was performed only with the OIR and OIS groups. Non-normally distributed (right-skewed) outcome variables were naturally log-transformed. Estimated β-effect sizes and 95% CIs were reported as the SD change in outcome variable per SD change in exposure variable, to facilitate direct comparisons of the strength of associations.

Statistical significance was set at *P* < .05 and the statistical analyses were performed in R version 4.2.0 (32).

## Results

### Characteristics of the Study Groups

Table 1 presents the characteristics of the study population. There was a tendency for older age and more girls in the OIR group (*P* = .016 for age; though nonsignificant, *P* = .102 for sex) than in OIS and NW groups. As expected, BMI SDS and waist–hip ratio were higher in subjects with obesity than in peers with NW; there was a tendency for higher BMI SDS in the OIR group than in the OIS group (though nonsignificant; *P* = .100). There was a higher proportion of individuals with IFG/IGT in the OIR group than in the OIS and NW groups (*P* = 3.62 × 10^–05^). The OIR group had higher historic fasting serum insulin concentrations than the OIS and NW groups (*P =* 2.59 × 10^–06^). The OIR group had higher plasma concentrations of HDL-C, TG, ALT, and GGT (all *P* < .05) than the OIS and NW groups (except for HDL-C *P* = .139 and GGT *P* = .133 in OIR vs OIS), with no significant difference between groups for AST (*P* = .111). The OIR group had higher HOMA-IR, lower SPISE, lower Matsuda index, higher IGI, and lower ODI than the OIS and NW groups (all *P* < .05; expect for ODI *P* = .170 in OIR vs NW).

**Table 1.** Clinical characteristics of the study groups

	OIR	OIS	NW	*P*	OIR vs OIS	OIR vs NW	OIS vs NW
N	22	22	36				
Age, years	13.6 (12.4, 15.3)	11.3 (9.4, 13.0)	11.2 (9.0, 13.3)	.016	.033	.021	.822
Sex (boys vs girls), n	6/16	13/9	15/21	.102	.109	.277	.277
BMI SDS	2.94 (2.73, 3.14)	2.63 (2.42, 3.01)	0.13 (0.01, 0.47)	1.21 × 10^–13^	.100	5.30 × 10^–16^	5.30 × 10^–16^
Waist-hip ratio	0.91 (0.86, 0.97)	0.89 (0.84, 0.94)	0.82 (0.79, 0.86)	1.79 × 10^–05^	.515	2.18 × 10^–04^	2.18 × 10^–04^
IFG/IGT vs NGT, n	12/10	1/21	4/32	3.62 × 10^–05^	5.79 × 10^–04^	5.79 × 10^–04^	.401
Historic insulin, pmol/L	214 (92, 243)	54 (41, 67)	44 (33, 64)	2.59 × 10^–06^	5.94 × 10^–04^	2.75 × 10^–07^	.120
HDL-C, mmol/L	1.00 (0.90, 1.17)	1.20 (1.00, 1.37)	1.40 (1.20, 1.60)	5.15 × 10^–04^	.139	3.86 × 10^–04^	.056
TG, mmol/L	1.45 (1.02, 1.80)	0.85 (0.55, 1.08)	0.60 (0.48, 0.80)	2.22 × 10^–07^	.001	1.60 × 10^–07^	.051
ALT, U/L	27.0 (22.3, 36.8)	22.5 (20.3, 25.8)	21.0 (18.0, 25.0)	.001	.030	.001	.174
AST, U/L	21.0 (20.0, 25.0)	23.0 (20.0, 27.5)	28.0 (20.0, 33.0)	.111	.432	.155	.283
GGT, U/L	19.5 (18.0, 20.8)	18.0 (16.0, 20.8)	15.5 (14.0, 17.0)	3.31 × 10^–05^	.133	2.76 × 10^–05^	.013
HOMA-IR	6.6 (4.7, 9.7)	1.8 (1.3, 2.7)	1.9 (1.4, 2.8)	1.29 × 10^–09^	7.75 × 10^–10^	8.64 × 10^–12^	.790
SPISE	6.4 (4.6, 7.5)	8.8 (7.3, 10.4)	15.3 (13.0, 17.8)	1.82 × 10^–13^	3.30 × 10^–04^	4.27 × 10^–15^	9.83 × 10^–12^
Matsuda index	1.9 (1.2, 2.5)	5.0 (3.6, 7.0)	5.2 (3.7, 8.1)	1.83 × 10^–09^	1.79 × 10^–10^	6.81 × 10^–12^	.616
IGI	2.7 (2.1, 3.4)	1.4 (1.1, 1.8)	1.1 (0.7, 1.8)	2.78 × 10^–06^	7.91 × 10^–05^	1.83 × 10^–06^	.141
ODI	0.11 (0.08, 0.12)	0.14 (0.12, 0.23)	0.12 (0.08, 0.16)	.021	.020	.170	.122

Continuous data are presented as medians (interquartile range; all cases considered non-normal). Kruskal–Wallis test for continuous variables and post hoc analyses using Wilcoxon rank sum tests. χ^2^ test for categorical variables. Bonferroni correction to adjust for pair-wise multiple testing (n = 3).

Abbreviations: ALT, alanine aminotransferase; AST, aspartate transaminase; GGT, γ-glutamyl transferase; HDL-C, high density lipoprotein cholesterol; HOMA-IR, homeostasis model assessment of insulin resistance; IFG, impaired fasting glucose; IGI, insulinogenic index; IGT, impaired glucose tolerance; NGT, normal glucose tolerance; NW, normal weight; ODI, oral disposition index; OIR, obesity and insulin resistance; OIS, obesity and insulin sensitive; SPISE, single-point insulin sensitivity estimator; TG, triglycerides.

### Plasma Glucose and Serum Insulin Response

**Figure 2.**
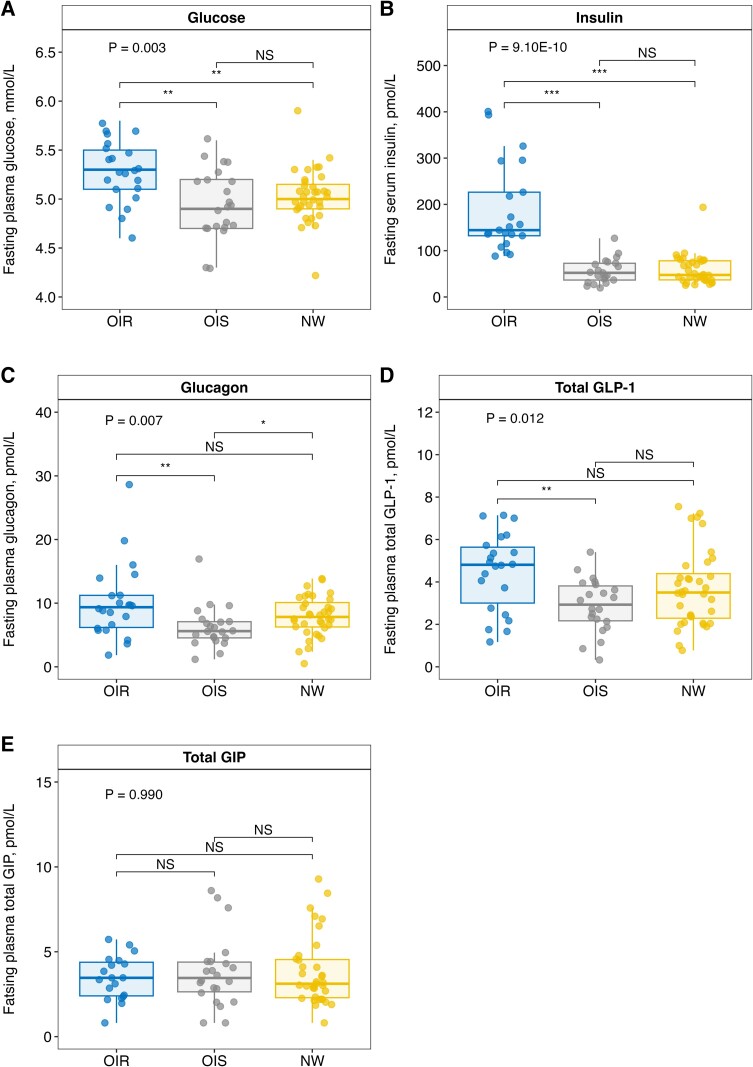
Fasting concentrations of (A) plasma glucose, (B) serum insulin, (C) plasma glucagon, (D) plasma total GLP-1, and (E) plasma total GIP during OGTT in children and adolescents with obesity and insulin resistance (OIR, n = 22), obesity and normal insulin sensitivity (OIS, n = 22), and controls with normal weight (NW, n = 36). *P* values for group differences calculated by Kruskal–Wallis test. Post hoc analyses using Wilcoxon rank sum tests with Bonferroni correction (n = 3). NS = not significant, *P < .05, **P < .01, ***P < .001.

Fasting concentrations of plasma glucose and serum insulin are shown in Fig. 2 and elsewhere (Table S1 (33)). Fasting concentrations of plasma glucose were higher in the OIR group than in the OIS (*P* = .007) and NW (*P* = .007) groups. As expected, fasting serum insulin concentrations were higher in the OIR group than in OIS (*P* = 2.51 × 10^–10^) and NW (*P* = 5.40 × 10^–12^) groups. There were no significant differences in fasting plasma glucose and serum insulin between the OIS and NW groups (*P* = .391; *P* = .854).

**Figure 3.**
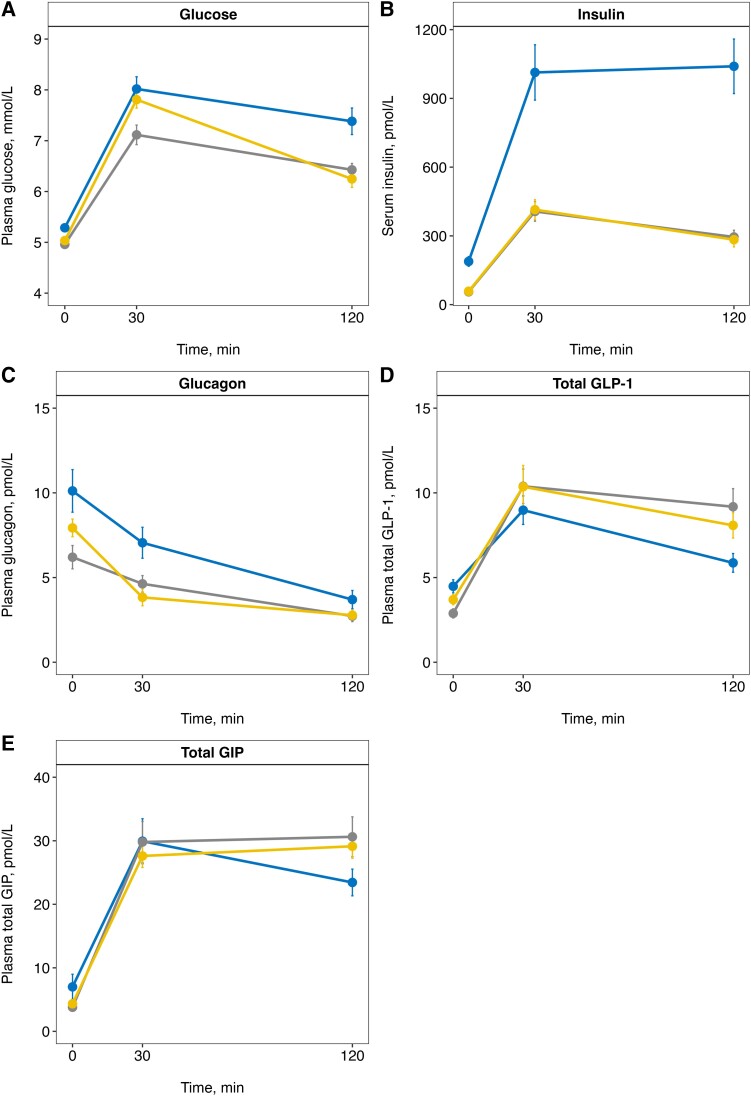
Concentrations of (A) plasma glucose, (B) serum insulin, (C) plasma glucagon, (D) plasma total GLP-1, and (E) plasma total GIP during an OGTT in children and adolescents with obesity and insulin resistance (OIR, blue, n = 22), obesity and normal insulin sensitivity (OIS, grey, n = 22), and controls with normal weight (NW, yellow, n = 36). Values are mean ± standard error of mean.

**Figure 4.**
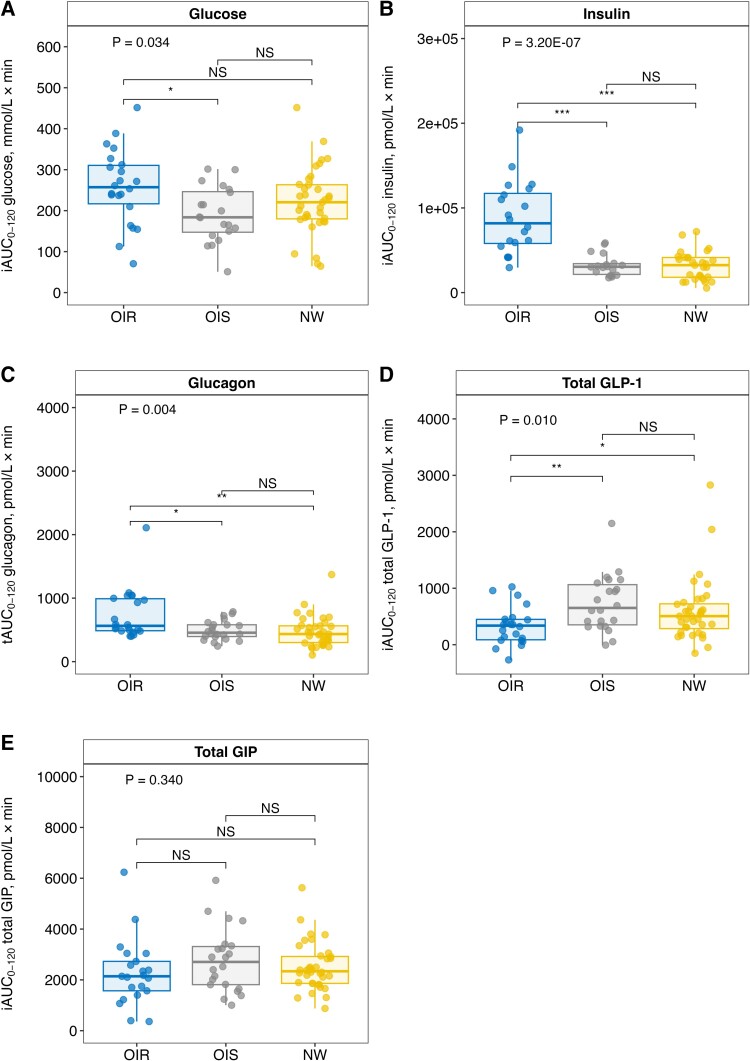
AUC for (A) plasma glucose (B) serum insulin, (C) plasma glucagon, (D) plasma total GLP-1, and (E) plasma total GIP during OGTT in children and adolescents with obesity and insulin resistance (OIR, n = 22), obesity and normal insulin sensitivity (OIS, n = 22), and controls with normal weight (NW, n = 36). *P* values for group differences calculated by Kruskal–Wallis test. Post hoc analyses using Wilcoxon rank sum tests with Bonferroni correction (n = 3). iAUC = incremental area under the curve, NS = not significant, tAUC = total area under the curve. *P < .05, **P < .01, ***P < .001.

Plasma glucose and serum insulin response during the OGTT are presented in Fig. 3 and elsewhere (Table S1 (33)). There was a tendency for higher plasma glucose (though nonsignificant) and higher serum insulin iAUC_0-120_ in the OIR group than in the OIS (*P* = .052; *P* = 8.22 × 10^–07^) and NW (*P* = .119; *P* = 1.28 × 10^–07^) groups. There was a trend for lower plasma glucose iAUC_0-120_ (though nonsignificant; *P* = .119) in the OIS compared with the NW group, with no significant difference in serum insulin iAUC_0-120_ (*P* = .803; Fig. 4; Table S1 (33)).

### Plasma Glucagon Response

Fasting concentrations of plasma glucagon are shown in Fig. 2 and elsewhere (Table S1 (33)). Fasting glucagon concentrations were higher in the OIR group than in the OIS (*P* = .014), but not for NW (*P* = .204) groups. Fasting glucagon concentrations were lower in the OIS than in the NW group (*P* = .019).

Plasma glucagon response during the OGTT is presented in Fig. 3 and elsewhere (Table S1 (33)). We observed a higher tAUC_0-120_ for glucagon in the OIR group than in the OIS (*P* = .017) and NW (*P* = .004) groups, with no significant differences between the OIS and NW groups (*P* = .377; Fig. 4; Table S1 (33)).

When the OIR group was stratified according to glucose tolerance status there was a trend for higher fasting glucagon (*P* = .114) and higher tAUC_0-120_ (*P* = .147) in individuals with IFG and/or IGT compared with normal glucose tolerance (NGT) (Fig. S1, Table S2 (33)).

### Plasma Total GLP-1 Response

Fasting concentrations of plasma total GLP-1 are shown in Fig. 2 and elsewhere (Table S1 (33)). Fasting GLP-1 concentrations were higher in the OIR group than in the OIS group (*P* = .007), with no significant differences between the OIR and NW (nonsignificant; *P* = .117), and OIS and NW groups (*P* = .135).

Plasma GLP-1 response during the OGTT is presented in Fig. 3 and elsewhere (Table S1 (33)). We observed a lower iAUC_0-120_ for GLP-1 in the OIR group than in the OIS (*P* = .018) and NW (*P* = .038) groups, with no significant differences between the OIS and NW groups (*P* = .153; Fig. 4, Table S1 (33)).

Individuals within the OIR group with IFG and/or IGT had higher fasting total GLP-1 (*P* = .010) and a trend for lower iAUC_0-120_ (nonsignificant; *P* = .114) than those with NGT (Fig. S1, Table S2 (33)).

### Plasma Total GIP Response

Fasting concentrations of plasma total GIP are shown in Fig. 2 and elsewhere (Table S1 (33)). No significant differences in fasting GIP were observed between study groups (*P* = .699).

Plasma GIP response during the OGTT is presented in Fig. 3 and elsewhere (Table S1 (33)). No significant differences in iAUC_0-120_ for GIP were observed between study groups (*P* = .227, Fig. 4, Table S1 (33)).

There were no significant differences in fasting total GIP (*P* = .356) and iAUC_0-120_ (*P* = .468) in individuals with IFG and/or IGT compared with NGT from the OIR group (Fig. S1, Table S2 (33)).

### Associations of Glucagon, GLP-1, and GIP With HOMA-IR

Higher fasting glucagon and a tendency for higher tAUC_0-120_ were associated with higher HOMA-IR (*P* = 2.52 × 10^–04^; *P* = .057) in a pooled model adjusted for age, sex, and BMI SDS (Table S3 (33)). Higher GLP-1 at fasting and lower iAUC_0-120_ were associated higher HOMA-IR (*P* = 1.34 × 10^–04^; *P* = .001). Lower GIP iAUC_0-120_ but not fasting GIP, was associated with higher HOMA-IR (*P* = .038; *P* = .479).

### Associations of Glucagon, GLP-1, and GIP With SPISE

Higher fasting glucagon and tAUC_0-120_ were associated with lower SPISE (*P* = .047; *P* = .033; Table S3 (33)). A lower iAUC_0-120_ showed a tendency for an association with lower SPISE, but not for fasting (nonsignificant; *P* = .056; *P* = .535). GIP at fasting and iAUC_0-120_ were not significantly associated with SPISE (*P* = .587; *P* = .656).

### Associations of Glucagon, GLP-1, and GIP With Matsuda Index

Higher fasting glucagon and tAUC_0-120_ were associated with a lower Matsuda index (*P* = 2.17 × 10^–05^; *P* = 4.36 × 10^–04^; Table S3 (33)). Higher fasting GLP-1 and lower iAUC_0-120_ were associated with a lower Matsuda index (*P* = 7.97 × 10^–05^; *P* = .028). Higher fasting GIP was associated with a higher Matsuda index, but not for iAUC_0-120_ (*P* = .031; *P* = .125).

### Associations of Glucagon, GLP-1, and GIP With IGI

Higher fasting glucagon and a tendency for higher tAUC_0-120_ was associated with higher IGI (*P* = .018; *P* = .098; Table S3 (33)). There was a tendency for higher fasting GLP-1 and higher IGI, but not for iAUC_0-120_ (nonsignificant; *P* = .086; *P* = .674). Fasting GIP and iAUC_0-120_ were not significantly associated with IGI (*P* = .850; *P* = .220).

### Associations of Glucagon, GLP-1, and GIP With ODI

Fasting glucagon and tAUC_0-120_ were not significantly associated with ODI (*P* = .132; *P* = .206; Table S3 (33)). Higher fasting GLP-1 and lower iAUC_0-120_ were associated with lower ODI (*P* = .044; *P* = .012). Higher fasting GIP was associated with lower ODI, but not for iAUC_0-120_ (*P* = .010; *P* = .372).

### Associations of Glucagon, GLP-1, and GIP With Liver Enzymes

Higher fasting glucagon was associated with higher fasting plasma concentrations of ALT, but not AST or GGT (*P* = .017; *P* = .407; *P* = .190; Table S3 (33)). Glucagon tAUC_0-120_ as well as fasting and iAUC_0-120_ for GLP-1 and GIP were not significantly associated with ALT, AST, or GGT (*P* ≥ .05).

Consistent results were observed in the subanalysis with OIR and OIS individuals only (Table S4 (33)) compared with the pooled model with all 3 study groups.

## Discussion

The present study reports that children and adolescents with OIR have elevated fasting concentrations of glucagon and GLP-1, with no significant difference in fasting GIP. Moreover, those with OIR exhibited elevated glucagon and attenuated GLP-1 responses during an OGTT compared with peers with OIS or NW. In contrast to children and adolescents with OIR, individuals with OIS display no significant differences in hormone responses compared with controls with NW. Elevated glucagon and attenuated GLP-1 responses were associated with worsened insulin sensitivity and β-cell function. Findings from this study suggest that insulin resistance is coupled to the obesity-related alterations in glucagon and GLP-1 secretion, which could have important implications for future disease risk.

Adults with obesity and T2D are characterized by elevated plasma concentrations of glucagon compared with healthy individuals, suggesting a potential resistance of the pancreatic α-cells to insulin (34-36). Altered glucagon response to glucose in adults has also been shown to independently associate with BMI and M values in EHC studies (37). Adolescents with obesity display similar elevations in fasting glucagon concentrations compared with controls with NW (7), associating with hyperinsulinemia, visceral adiposity, high plasma free fatty acids, plasma triglycerides, and IGT (9). Inadequate suppression of glucagon during an EHC also occurs in adolescents with obesity and IGT (8). In the present study, we are able to delineate that children and adolescents with obesity and insulin resistance have elevated concentrations of glucagon at fasting and higher glucagon responses during an OGTT compared with individuals with obesity and normal insulin sensitivity, and controls with NW.

Adults with obesity and T2D have lower GLP-1 responses after an oral glucose load than healthy individuals, which associates with worsened insulin sensitivity and impaired β-cell function (38). Young adults with obesity, higher liver fat content, and hepatic insulin resistance show a similar attenuated GLP-1 response to oral glucose compared with healthy controls (39). Several prior studies in children and adolescents with obesity report blunted GLP-1 responses compared with controls with NW (10-12), suggesting that altered GLP-1 secretion emerges early in life. In addition to lower GLP-1 responses, elevated fasting GLP-1 has been reported in children and adolescents with obesity compared with NW peers (10, 11, 13, 15, 16). Interestingly, higher fasting concentrations and lower GLP-1 response to an oral glucose challenge has also been observed in a rodent model of chronic hyperinsulinemia (MKR mice) compared with control mice (40). In agreement with prior evidence, the present study finds that children and adolescents with obesity and insulin resistance exhibit higher fasting concentrations of GLP-1 and lower GLP-1 responses to oral glucose than peers with obesity and normal insulin sensitivity, and controls with NW, associating with lower insulin sensitivity and impaired β-cell function. Alterations in GLP-1 responses may also further deviate according to glucose tolerance status.

Similar to GLP-1, impaired GIP secretion is also expected in adults with obesity and T2D (41). Yet, a large meta-analysis found no significant differences in GIP secretion between adult T2D patients (n = 363) and subjects with NGT (n = 325) (42). Likewise, results from the large-scale ADDITION-PRO study (n = 1405) revealed no significant differences in GIP response during an OGTT according to glucose tolerance status in adults (43). Interestingly, higher fasting GIP was associated with improved lipid metabolism (ie, lower LDL-C in bith men and women, and higher HDL-C in women), whereas higher stimulated GIP was associated with unhealthy fat distribution (ie, more visceral abdominal fat and higher waist to hip ratio) in men, independent of insulin (43). Previous studies in children and adolescents with obesity also found no significant differences in GIP response during OGTT or EHC (15-18). In the present study we found no significant differences in GIP response to oral glucose in children and adolescents with obesity and insulin resistance. Based on the present findings, the role of GIP in pediatric obesity still remains unclear. Future studies are needed to dissect the associations (if any) between changes in body fat distribution and insulin resistance on perturbed GIP secretion.

Further support for the relationships found in the present study comes from adults in the IMI-DIRECT consortium (n = 726), which applied soft clustering of clinical phenotypes, exposing an archetype of obesity and insulin resistance associated with higher visceral fat and liver fat, lower physical activity, and higher fasting concentrations of glucagon and GLP-1 (44). In the present study, children and adolescents with obesity but normal insulin sensitivity seem to be protected against alterations in glucagon and incretin secretion compared to individuals with insulin resistance. Yet, despite this lack of difference in hormone levels in the OIS group, evidence suggest that adults with OIS have elevated cardiometabolic risk compared with those with NW and normal insulin sensitivity (45).

Heterogeneity in fat distribution may play a key role in the relationship between insulin resistance and altered hormone secretion. The adipose expandability theory serves to explain the individual variability in the upper level of fat storage capacity, which when exceeded elicits ectopic fat storage in visceral depots such as in the liver and skeletal muscles, contributing to insulin resistance and low-grade chronic inflammation (46).

The concept of a liver–α cell axis may serve as a possible mechanism, linking obesity, insulin resistance, and hyperglucagonemia. According to this theory, increased fat in the liver impairs glucagon signaling leading to a decrease in ureagenesis, an increase in plasma amino acids, in turn increasing glucagon secretion from the α-cells causing hyperglucagonemia (36). The glucagon–alanine index has been proposed as a biomarker of liver–α cell axis, as it associates with higher hepatic insulin resistance, plasma concentrations of ALT and GGT in the ADDITION-PRO study (47). A similar liver–α cell axis may exist in children and adolescents with obesity and insulin resistance (48). The current study found a positive associations between fasting glucagon and fasting plasma concentrations of ALT, but not with GLP-1 or GIP.

There are several strengths and limitations that apply to the present cross-sectional study. One limitation is the lack of information on pubertal stage and measurement of sex hormones, as transient physiological insulin resistance occurs during growth and development, which may limit the interpretations of our findings (23). This study included 0, 30, and 120 minute time points. Additional time points (eg, 180 minutes) during the OGTT could enhance the power to detect differences in hormone responses between groups (49). The children and adolescents with obesity included in this study were undergoing an intervention program at an obesity treatment clinic (20), which may have affected measures of glucose metabolism and hormone concentrations beneficially, potentially limiting the power to detect significant differences. This consideration does not apply to the participants with NW, who were not part of an intervention program. Moreover, we did not perform the more laborious EHC, which is the gold standard for assessing insulin sensitivity (50). Instead, we used surrogate measures, including the Matsuda index, HOMA-IR, and SPISE, which show a moderate degree of correlation in children and adolescents with obesity (27, 51). We also included the IGI as a correlate for acute insulin response (30) and ODI as an informative estimate of β-cell function in children and adolescents with obesity (52). A major strength to the present study is the use of well-documented and validated assays for plasma glucagon and GLP-1 with high specificity and sensitivity (53-55). The recruitment of subjects stratified by insulin sensitivity allowed us to uniquely examine the combined effects of obesity and insulin resistance on glucagon, GLP-1, and GIP secretion, which has not been previously characterized in pediatric studies.

Due to the cross-sectional nature of this study, we cannot conclude whether altered hormone secretion precedes or parallels changes in insulin resistance. It also remains unclear whether insulin resistance in children and adolescents with obesity is a transient phenotype or whether it progresses over time. Future longitudinal studies are warranted to examine whether altered glucagon and GLP-1 secretion in children and adolescents with obesity and insulin resistance increase the risk of diabetes development into adulthood. Moreover, application of Mendelian randomization techniques might elucidate the (suggestive) causal relationship between obesity, insulin resistance, and altered glucagon and gut hormone secretion.

## Conclusions

Children and adolescents with obesity and insulin resistance have elevated concentrations of fasting plasma glucagon and GLP-1, elevated glucagon, and attenuated GLP-1 responses during the OGTT, which is paralleled by lower estimates of insulin sensitivity and altered β-cell function. Children and adolescents with obesity and normal insulin sensitivity do not exhibit similar alterations in glucagon and incretin secretion, highlighting potential for targeted interventions.

## Data Availability

Restrictions apply to the availability of some, or all, data generated or analyzed during this study to preserve patient confidentiality. The corresponding author will on request detail the restrictions and any conditions under which access to some data may be provided.

## Abbreviations

ALT: alanine transaminase
AST: aspartate transaminase
BMI: body mass index
EHC: euglycemic–hyperinsulinemic clamp
GGT: γ-glutamyl transferase
GIP: glucose-dependent insulinotropic polypeptide
GLP-1: glucagon-like peptide-1
HDL-C: high-density lipoprotein cholesterol
HOMA-IR: homeostatic model assessment of insulin resistance
iAUC: incremental area under the curve
IFG: impaired fasting glucose
IGI: insulinogenic index
IGT: impaired glucose tolerance
NGT: normal glucose tolerance
NW: normal weight
ODI: oral disposition index
OGTT: oral glucose tolerance test
OIR: obesity and insulin resistance
OIS: obesity and normal insulin sensitivity
SDS: standard deviation score
SPISE: single-point insulin sensitivity estimator
tAUC: total area under the curve
T2D: type 2 diabetes
TG: triglyceride

## References

[1] Jebeile H, Kelly AS, O'Malley G, Baur LA. Obesity in children and adolescents: epidemiology, causes, assessment, and management. Lancet Diabetes Endocrinol. 2022;10(5):351‐365. 35248172 10.1016/S2213-8587(22)00047-X PMC9831747

[2] Weiss R, Kaufman FR. Metabolic complications of childhood obesity: identifying and mitigating the risk. Diabetes Care. 2008;31(Suppl 2):S310‐S316. 18227502 10.2337/dc08-s273

[3] Stefan N . Causes, consequences, and treatment of metabolically unhealthy fat distribution. Lancet Diabetes Endocrinol. 2020;8(7):616‐627. 32559477 10.1016/S2213-8587(20)30110-8

[4] Muller TD, Finan B, Clemmensen C, DiMarchi RD, Tschop MH. The new biology and pharmacology of glucagon. Physiol Rev. 2017;97(2):721‐766. 28275047 10.1152/physrev.00025.2016

[5] Del Prato S, Gallwitz B, Holst JJ, Meier JJ. The incretin/glucagon system as a target for pharmacotherapy of obesity. Obes Rev. 2022;23(2):e13372. 34713962 10.1111/obr.13372 PMC9286339

[6] Stinson SE, Jonsson AE, de Retana Alzola IF, et al Hyperglucagonemia in pediatric adiposity associates with cardiometabolic risk factors but not hyperglycemia. J Clin Endocrinol Metab. 2022;107(6):1569‐1576. 35213713 10.1210/clinem/dgac108 PMC9113783

[7] Lat J, Caprio S. Understanding the pathophysiology of youth-onset Type 2 Diabetes (T2D): importance of alpha-cell function. J Clin Endocrinol Metab. 2022;107(9):e3957‐e3958. 35512384 10.1210/clinem/dgac273 PMC9387691

[8] Weiss R, D'Adamo E, Santoro N, Hershkop K, Caprio S. Basal alpha-cell up-regulation in obese insulin-resistant adolescents. J Clin Endocrinol Metab. 2011;96(1):91‐97. 20843946 10.1210/jc.2010-1275 PMC3038472

[9] Manell H, Kristinsson H, Kullberg J, et al Hyperglucagonemia in youth is associated with high plasma free fatty acids, visceral adiposity, and impaired glucose tolerance. Pediatr Diabetes. 2019;20(7):880‐891. 31271247 10.1111/pedi.12890

[10] Stenlid R, Manell H, Halldin M, et al High DPP-4 concentrations in adolescents are associated with low intact GLP-1. J Clin Endocrinol Metab. 2018;103(8):2958‐2966. 29850829 10.1210/jc.2018-00194

[11] Higgins V, Asgari S, Hamilton JK, et al Postprandial dyslipidemia, hyperinsulinemia, and impaired gut peptides/bile acids in adolescents with obesity. J Clin Endocrinol Metab. 2020;105(4):1228‐1241. 31825485 10.1210/clinem/dgz261 PMC7065844

[12] Galderisi A, Giannini C, Van Name M, Caprio S. Fructose consumption contributes to hyperinsulinemia in adolescents with obesity through a GLP-1-mediated mechanism. J Clin Endocrinol Metab. 2019;104(8):3481‐3490. 30938760 10.1210/jc.2019-00161 PMC6599430

[13] Stinson SE, Jonsson AE, Lund MAV, et al Fasting plasma GLP-1 is associated with overweight/obesity and cardiometabolic risk factors in children and adolescents. J Clin Endocrinol Metab. 2021;106(6):1718‐1727. 33596309 10.1210/clinem/dgab098 PMC8118577

[14] Kubota S, Yabe D. Elevation of fasting GLP-1 levels in child and adolescent obesity: friend or foe? J Clin Endocrinol Metab. 2021;106(9):e3778‐e3780. 33950185 10.1210/clinem/dgab301 PMC8372656

[15] Michaliszyn SF, Mari A, Lee S, et al beta-cell function, incretin effect, and incretin hormones in obese youth along the span of glucose tolerance from normal to prediabetes to type 2 diabetes. Diabetes. 2014;63(11):3846‐3855. 24947360 10.2337/db13-1951 PMC4207396

[16] Aulinger BA, Vahl TP, Prigeon RL, D’Alessio DA, Elder DA. The incretin effect in obese adolescents with and without type 2 diabetes: impaired or intact? Am J Physiol Endocrinol Metab. 2016;310(9):E774‐E781. 26979523 10.1152/ajpendo.00496.2015 PMC4867309

[17] Sakornyutthadej N, Mahachoklertwattana P, Chanprasertyothin S, Pongratanakul S, Khlairit P, Poomthavorn P. Beta cell function, incretin hormones, and incretin effect in obese children and adolescents with prediabetes. Pediatr Diabetes. 2022;23(2):203‐211. 34913553 10.1111/pedi.13303

[18] Heptulla RA, Tamborlane WV, Cavaghan M, et al Augmentation of alimentary insulin secretion despite similar gastric inhibitory peptide (GIP) responses in juvenile obesity. Pediatr Res. 2000;47(5):628‐633. 10813588 10.1203/00006450-200005000-00012

[19] Holst JJ . Glucagon and other proglucagon-derived peptides in the pathogenesis of obesity. Front Nutr. 2022;9:964406. 35990325 10.3389/fnut.2022.964406 PMC9386348

[20] Holm JC, Gamborg M, Bille DS, Gr Nb KH, Ward LC, Faerk J. Chronic care treatment of obese children and adolescents. Int J Pediatr Obes. 2011;6(3-4):188‐196. 21529264 10.3109/17477166.2011.575157

[21] Lausten-Thomsen U, Christiansen M, Fonvig CE, et al Reference values for serum total adiponectin in healthy non-obese children and adolescents. Clin Chim Acta. 2015;450:11‐14. 26169157 10.1016/j.cca.2015.07.012

[22] American Diabetes A . 2. Classification and diagnosis of diabetes: standards of medical care in diabetes-2021. Diabetes Care. 2021;44(Suppl 1):S15‐S33. 33298413 10.2337/dc21-S002

[23] Frithioff-Bojsoe C, Lund MAV, Kloppenborg JT, et al Glucose metabolism in children and adolescents: population-based reference values and comparisons to children and adolescents enrolled in obesity treatment. Pediatr Diabetes. 2019;20(5):538‐548. 31074070 10.1111/pedi.12859

[24] Nysom K, Molgaard C, Hutchings B, Michaelsen KF. Body mass index of 0 to 45-y-old Danes: reference values and comparison with published European reference values. Int J Obes Relat Metab Disord. 2001;25(2):177‐184. 11410817 10.1038/sj.ijo.0801515

[25] Frithioff-Bojsoe C, Trier C, Esmann Fonvig C, et al Estimates of insulin sensitivity and beta-cell function in children and adolescents with and without components of the metabolic syndrome. Pediatr Endocrinol Diabetes Metab. 2017;23(3):122‐129. 29253032 10.18544/PEDM-23.03.0083

[26] Matthews DR, Hosker JP, Rudenski AS, Naylor BA, Treacher DF, Turner RC. Homeostasis model assessment: insulin resistance and beta-cell function from fasting plasma glucose and insulin concentrations in man. Diabetologia. 1985;28(7):412‐419. 3899825 10.1007/BF00280883

[27] Paulmichl K, Hatunic M, Hojlund K, et al Modification and validation of the triglyceride-to-HDL cholesterol ratio as a surrogate of insulin sensitivity in white juveniles and adults without diabetes Mellitus: the single point insulin sensitivity estimator (SPISE). Clin Chem. 2016;62(9):1211‐1219. 27471037 10.1373/clinchem.2016.257436

[28] Matsuda M, DeFronzo RA. Insulin sensitivity indices obtained from oral glucose tolerance testing: comparison with the euglycemic insulin clamp. Diabetes Care. 1999;22(9):1462‐1470. 10480510 10.2337/diacare.22.9.1462

[29] Seltzer HS, Allen EW, Herron AL Jr, Brennan MT. Insulin secretion in response to glycemic stimulus: relation of delayed initial release to carbohydrate intolerance in mild diabetes mellitus. J Clin Invest. 1967;46(3):323‐335. 6023769 10.1172/JCI105534 PMC297053

[30] Phillips DI, Clark PM, Hales CN, Osmond C. Understanding oral glucose tolerance: comparison of glucose or insulin measurements during the oral glucose tolerance test with specific measurements of insulin resistance and insulin secretion. Diabet Med. 1994;11(3):286‐292. 8033528 10.1111/j.1464-5491.1994.tb00273.x

[31] Utzschneider KM, Prigeon RL, Faulenbach MV, et al Oral disposition index predicts the development of future diabetes above and beyond fasting and 2-h glucose levels. Diabetes Care. 2009;32(2):335‐341. 18957530 10.2337/dc08-1478 PMC2628704

[32] R Core Team . R: A Language and Environment for Statistical Computing. R Foundation for Statistical Computing; 2021.

[33] Stinson SE, Fernández de Retana Alzola I, Damgaard Brünner Hovendal E, et al Altered glucagon and GLP-1 responses to oral glucose in children and adolescents with obesity and insulin resistance. J Clin Endocrinol Metab. 2024;109(6):1590‐1600. 10.1210/clinem/dgad728 PMC11099488 38087928

[34] Ahren B, Larsson H. Impaired glucose tolerance (IGT) is associated with reduced insulin-induced suppression of glucagon concentrations. Diabetologia. 2001;44(11):1998‐2003. 11719830 10.1007/s001250100003

[35] Faerch K, Vistisen D, Pacini G, et al Insulin resistance is accompanied by increased fasting glucagon and delayed glucagon suppression in individuals with normal and impaired glucose regulation. Diabetes. 2016;65(11):3473‐3481. 27504013 10.2337/db16-0240

[36] Wewer Albrechtsen NJ, Pedersen J, Galsgaard KD, et al The liver-alpha-cell axis and type 2 diabetes. Endocr Rev. 2019;40(5):1353‐1366. 30920583 10.1210/er.2018-00251

[37] Lundqvist MH, Almby K, Wiklund U, et al Altered hormonal and autonomic nerve responses to hypo- and hyperglycaemia are found in overweight and insulin-resistant individuals and may contribute to the development of type 2 diabetes. Diabetologia. 2021;64(3):641‐655. 33241460 10.1007/s00125-020-05332-z PMC7864814

[38] Faerch K, Torekov SS, Vistisen D, et al GLP-1 Response to oral glucose is reduced in prediabetes, screen-detected type 2 diabetes, and obesity and influenced by sex: the ADDITION-PRO study. Diabetes. 2015;64(7):2513‐2525. 25677912 10.2337/db14-1751

[39] Matikainen N, Bogl LH, Hakkarainen A, et al GLP-1 responses are heritable and blunted in acquired obesity with high liver fat and insulin resistance. Diabetes Care. 2014;37(1):242‐251. 23990519 10.2337/dc13-1283

[40] Lim GE, Huang GJ, Flora N, LeRoith D, Rhodes CJ, Brubaker PL. Insulin regulates glucagon-like peptide-1 secretion from the enteroendocrine L cell. Endocrinology. 2009;150(2):580‐591. 18818290 10.1210/en.2008-0726 PMC5393261

[41] Nauck MA, Meier JJ. Incretin hormones: their role in health and disease. Diabetes Obes Metab. 2018;20(Suppl 1):5‐21. 29364588 10.1111/dom.13129

[42] Calanna S, Christensen M, Holst JJ, et al Secretion of glucose-dependent insulinotropic polypeptide in patients with type 2 diabetes: systematic review and meta-analysis of clinical studies. Diabetes Care. 2013;36(10):3346‐3352. 24065842 10.2337/dc13-0465 PMC3781498

[43] Moller CL, Vistisen D, Faerch K, et al Glucose-Dependent insulinotropic polypeptide is associated with lower low-density lipoprotein but unhealthy fat distribution, independent of insulin: the ADDITION-PRO study. J Clin Endocrinol Metab. 2016;101(2):485‐493. 26505824 10.1210/jc.2015-3133

[44] Wesolowska-Andersen A, Brorsson CA, Bizzotto R, et al Four groups of type 2 diabetes contribute to the etiological and clinical heterogeneity in newly diagnosed individuals: an IMI DIRECT study. Cell Rep Med. 2022;3(1):100477. 35106505 10.1016/j.xcrm.2021.100477 PMC8784706

[45] Hoddy KK, Axelrod CL, Mey JT, et al Insulin resistance persists despite a metabolically healthy obesity phenotype. Obesity (Silver Spring). 2022;30(1):39‐44. 34816598 10.1002/oby.23312 PMC9136885

[46] Buemann B, Sorensen TI, Pedersen O, et al Lower-body fat mass as an independent marker of insulin sensitivity--the role of adiponectin. Int J Obes (Lond). 2005;29(6):624‐631. 15824752 10.1038/sj.ijo.0802929

[47] Wewer Albrechtsen NJ, Faerch K, Jensen TM, et al Evidence of a liver-alpha cell axis in humans: hepatic insulin resistance attenuates relationship between fasting plasma glucagon and glucagonotropic amino acids. Diabetologia. 2018;61(3):671‐680. 29305624 10.1007/s00125-017-4535-5

[48] Gar C, Haschka SJ, Kern-Matschilles S, et al The liver-alpha cell axis associates with liver fat and insulin resistance: a validation study in women with non-steatotic liver fat levels. Diabetologia. 2021;64(3):512‐520. 33275161 10.1007/s00125-020-05334-x PMC7864806

[49] Halloun R, Galderisi A, Caprio S, Weiss R. Lack of evidence for a causal role of hyperinsulinemia in the progression of obesity in children and adolescents: a longitudinal study. Diabetes Care. 2022;45(6):1400‐1407. 35235641 10.2337/dc21-2210 PMC9210872

[50] DeFronzo RA, Tobin JD, Andres R. Glucose clamp technique: a method for quantifying insulin secretion and resistance. Am J Physiol. 1979;237(3):E214‐E223. 382871 10.1152/ajpendo.1979.237.3.E214

[51] Yeckel CW, Weiss R, Dziura J, et al Validation of insulin sensitivity indices from oral glucose tolerance test parameters in obese children and adolescents. J Clin Endocrinol Metab. 2004;89(3):1096‐1101. 15001593 10.1210/jc.2003-031503

[52] Caprio S . The oral disposition index: a valuable estimate of beta-cell function in obese youth. J Pediatr. 2012;161(1):3‐4. 22464745 10.1016/j.jpeds.2012.02.013

[53] Bak MJ, Wewer Albrechtsen NJ, Pedersen J, et al Specificity and sensitivity of commercially available assays for glucagon-like peptide-1 (GLP-1): implications for GLP-1 measurements in clinical studies. Diabetes Obes Metab. 2014;16(11):1155‐1164. 25041349 10.1111/dom.12352

[54] Wewer Albrechtsen NJ, Veedfald S, Plamboeck A, et al Inability of some commercial assays to measure suppression of glucagon secretion. J Diabetes Res. 2016;2016:8352957. 26839899 10.1155/2016/8352957 PMC4709665

[55] Wewer Albrechtsen NJ, Hartmann B, Veedfald S, et al Hyperglucagonaemia analysed by glucagon sandwich ELISA: nonspecific interference or truly elevated levels? Diabetologia. 2014;57(9):1919‐1926. 24891019 10.1007/s00125-014-3283-z

